# The effect of hydroalcoholic extract of Achillea eriophora DC. on blood pressure of anaesthetized male rat

**DOI:** 10.17179/excli2016-529

**Published:** 2016-12-07

**Authors:** Sohrab Anvari, Aminollah Bahaoddini, Mahmoodreza Moein, Ahmad Reza Khosravi

**Affiliations:** 1 Department of Biology, College of Sciences, Shiraz University, Shiraz, Iran; 2 Department of Pharmacognosy, School of Pharmacy, Shiraz University of Medical Sciences, Shiraz, Iran

**Keywords:** Achillea eriophora, hypotension, cholinergic system, nitrergic system

## Abstract

*Achillea eriophora *(Asteraceae) is a medicinal plant commonly used in Iran. This study was performed to determine the cardiovascular effects of hydroethanolic extract of *A*. *eriophora *(HEAE) and the underlying mechanisms in anaesthetized rats. The acute effects of intravenous (i.v.) administration of different doses of HEAE (40, 50, 60, 80 mg/kg), and its probable interaction with cholinergic and nitrergic systems were investigated in the presence of ACh and NOS blocker (L-NAME) as well as ethanol (HEAE solvent in sham group). Intravenous administration of different doses of HEAE induced hypotension. HEAE (60 mg/kg) significantly reduced mean arterial blood pressure (MAP), systolic arterial blood pressure (SBP) and diastolic arterial blood pressure (DBP) compared to control rats that treated with ethanol only, but no change in heart rate (HR) was seen in both groups. The results showed significant decrease in MAP, SBP, DBP and increase of HR in the presence of HEAE plus ACh (10 µg/kg) compared to when ACh was injected alone. Finally i.v. administration of HEAE, significantly reduced MAP and DBP in L-NAME (5 mg/kg) treated animals, while bradycardic responses to L-NAME were not significantly changed by HEAE. It can be concluded that *Achillea eriophora* induced hypotensive effect via lowering total peripheral resistance and cardiac output that may be synergist with cholinergic and independent of nitrergic system.

## Introduction

*Achillea eriophora* DC. is an endemic species of *Achillea* genus in Iran. This plant is growing mainly in the southern provinces of Iran at an altitude of 700-3000 m (Weyerstahl et al., 1997[28]). In Iran, it is popularly known as “Bumadaran-e Shiraz” or “Sarzardu” and commonly used in traditional medicine for treatment of gastrointestinal disorders (Zargari, 1968[32]). *A. eriophora *and related species are also used in traditional Persian medicine against various liver diseases, dysmenorrhea, inflammation, fever and cardiovascular disorders (Zargari, 1968[32]; 1984[31]). The genus *Achillea *L. belongs to Asteraceae, the largest family of vascular plants. Several species of *Achillea* have been used in folk medicine as anti-inflammatory, antispasmodic, diaphoretic and diuretic agents and for treatment of hemorrhagic side effects, pneumonia, rheumatic pain, and wounds since past centuries all over the world (Karamenderes and Apaydin, 2003[14]; Si et al., 2006[25]; Yaeesh et al., 2006[30]; De Souza et al., 2013[6]).

Among different species of the genus, *A. millefolium *L. have been tested in numerous experimental studies (reviewed in Nemeth and Bernath, 2008[20]). In the previous studies, cardiovascular effects of this species such as hypotensive effect (DeSouza et al., 2011[7]) and vasoprotective activity (Dall'Acqua et al., 2011[5]) have been reported. Other species, *A. wilhelmsii* C. Koch is one of the wide spread species of *Achillea* in Iran (Kazemi and Rostami, 2015[15]). It is shown that this plant has antioxidant and antimicrobial (Kazemi and Rostami, 2015[15]), immunomodulatory (Sharififar et al., 2009[23]) and antimycobacterial (Tosun et al., 2004[26]) activity. Furtheremore, it has been studied for its preventive role in hepatotoxicity (Dadkhah et al., 2014[4]), inhibitory effect on gastric acid output (Niazmand et al., 2010[21]), antihypertensive and antihyperlipidemic effects (Asgary et al., 2000[2]).

Phytochemical investigations of *Achillea* species revealed the presence of various bioactive constituents. In the previous studies, the flavonoids, terpenoids, lignans, aminoacid derivatives, fatty acids and alkamids have been identified in *Achillea* species (reviewed in Si et al., 2006[25]). Despite the folkloric use, there are a few reports dealing with the chemical composition of *A. eriophora *and its biological activities (e.g. Weyerstahl et al., 1997[28]; Ghasemi et al., 2008[10]). Ghasemi et al. (2008[10]) reported thirty-two chemical components from *A. eriophora* essential oil, such as 1,8 cineole, linalool, α-terpineole, and geranylformate. Considering the previous studies (e.g. Asgary et al., 2000[2]; Dall'Acqua et al., 2011[5]; De Souza et al., 2011[7]) about cardiovascular effects of different species of *Achillea *genus and no scientific and direct data of cardiovascular effects of the species *A*. *eriophora*, the present study was done. It was supposed to determine more clear and complementary information about cardiovascular effects of hydroethanolic extract obtained from leaves and flowers of *A*. *eriophora*. 

### Chemical compounds studied in this article

Ethanol (PubChem CID: 702); Urethane (PubChem CID: 5641); Heparin Sodium (PubChem CID: 22833565); Acetylcholine Chloride (PubChem CID: 6060); L-NAME (PubChem CID: 39836)

## Materials and Methods

Male wistar rats (220-250 g) were used in all experiments and housed at 22 ± 2 ºC under 12-hour light/12-hour dark cycle with free access to food and water. The protocols and procedures were approved by the Shiraz University Ethics Committee.

### Preparation of the extract

Material of *Achillea eriophora *was collected in June 2013 from Shiraz mountain, Fars province of Iran at 1600 m altitude above sea level ( N 29º 38΄ 52 - E 52º 31΄ 2). The plant was identified by A.R. Khosravi from Herbarium of Shiraz University (HSU). The voucher (no. 25049) were prepared and deposited in the HSU. Dried and powdered leaves and flowers of *A. eriophora* were extracted with 70 % ethanol. The hydroethanolic extract was concentrated using rotary evaporator. The concentrated extract was freez-dried and the powder (yield 16.36 % w/w of dried plant material) was freshly dissolved in ethanol (70 %) before administration.

### Surgical preparations

Adult male wistar rats were anaesthetized by intraperitoneal injection of urethane (1.2 g/kg). The animals were allowed to breath spontaneously through a tracheostomy. The left femoral vein and artery were exposed and cannulated with a heparinized polyethylene catheter (PE-50). The vein cannula was used for i.v. injection of the drugs, extract and extract solvent (ethanol 70 %), while the arterial cannula filled with heparinized saline and connected to a pressure transducer (MLT844), which was connected to a powerlab (ADInstruments Company, Australia) for arterial blood pressure and heart rate recording. Immediately after venous cannulation, the rats were injected with heparin (30 IU), to prevent blood clotting. In order to stabilization of the blood pressure after the surgical process, an interval of 60 min was held before any recording. During experiment, rectal temperature was kept close to 37˚C and 5 % dextrose in normal saline was i.v. injected (3 ml/h).

### Experimental protocol 

Four series of experiments were performed as follows.

#### Series 1

**Figure 1:**
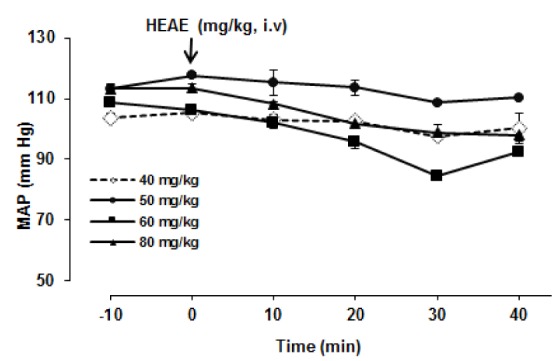
Effects of intravenous injection of different doses of HEAE on the mean arterial blood pressure (MAP) of anaesthetized rats. The treatment started at min zero. Data are means ± S.E.M. of four animals

In the first series of experiments, different doses of HEAE (40, 50, 60, 80 mg/kg) were i.v. administrated after the stabilization of blood pressure and HR. Each doses of HEAE was evaluated in four animals. The HEAE (60 mg/kg), defined as effective dose that produced immediate and long-lasting changes in MAP compared to the baseline values (Figure 1(Fig. 1)).

#### Series 2

In the second series, blood pressure and HR were recorded in a period of 30 min, then HEAE (60 mg/kg, i.v.) or equivalent volume of its solvent (ethanol 70 %, i.v.) were injected to rats (n = 5 for each treatment) and arterial blood pressure (MAP, SBP, DBP) and HR were continuously monitored for a period of 30 min.

#### Series 3

This series of experiments was performed to assess the interaction of HEAE and its solvent (ethanol 70 %) with cholinergic system by the following procedure: first group served as experimental group in which distilled water, ACh, HEAE+ACh were injected respectively (n = 5). The second group served as sham operated group in which distilled water, ACh, ethanol+ACh were injected respectively (n = 5). In both groups, after the blood pressure stabilization, distilled water (as ACh solvent) was injected and cardiovascular parameters such as HR, MAP, etc. were recorded for 10 min, then ACh (10 µg/kg, i.v.) was injected and its effects on above parameters were recorded. Cardiovascular parameters were allowed to return to baseline levels. In experimental group, in the last stage, HEAE (60 mg/kg) was injected and after the onset of the hypotension (after the fifth min), the rats received ACh and consequently, the parameters were recorded. In sham operated group, in the last stage, ethanol (equivalent volume of HEAE) was injected and after the fifth min, the animals received ACh and their cardiovascular parameters were recorded.

#### Series 4

This series of experiments was performed to determine the interaction of HEAE and its solvent (ethanol 70 %) with nitrergic system by the following procedure: the first group as experimental group, in which distilled water (as L-NAME solvent), L-NAME (5mg/kg), HEAE (60 mg/kg) were injected respectively (n = 5). The second group as sham operated group, in which distilled water, L-NAME (5mg/kg), ethanol (equivalent volume of HEAE) were injected respectively (n = 5). 

In the whole above series, the cardiovascular parameters were recorded at the same times for sham and experimental rats. Acetylcholine Chloride (Sigma-Aldrich Co., St. Louis, MO, USA) and L-NAME (Sigma-Aldrich Co., St. Louis, MO, USA) were dissolved in distilled water and freshly prepared before any experiment. All drugs, extract and solvent were injected similarly to sham operated and experimental groups.

### Calculation and statistical analysis

The data are expressed as the mean value ± standard error of the mean (S.E.M). Significant difference between groups was determined with Student's t-test, and difference between stages of one group was determined with repeated measure or paired sample t-test using SPSS software (version 21.0). In all analysis, a p value < 0.05 considered as statistically significant.

## Results

### Effects of different doses of hydroethanolic extract of A. eriophora (HEAE) on mean arterial blood pressure (MAP).

After the stabilization of blood pressure, i.v. administration of different doses of HEAE (40, 50, 60, 80 mg/kg) in different rats decreased MAP values (Figure 1(Fig. 1)). This effect became significant and long-lasting at the dose of 60 mg/kg, compared with the HEAE preinjection MAP values. This dose is close to the maximum immediate hypotensive effect and this effect lasted about 60 min after treatment.

### Effects of HEAE and its solvent (ethanol 70 %) on arterial blood pressure and HR

**Figure 2:**
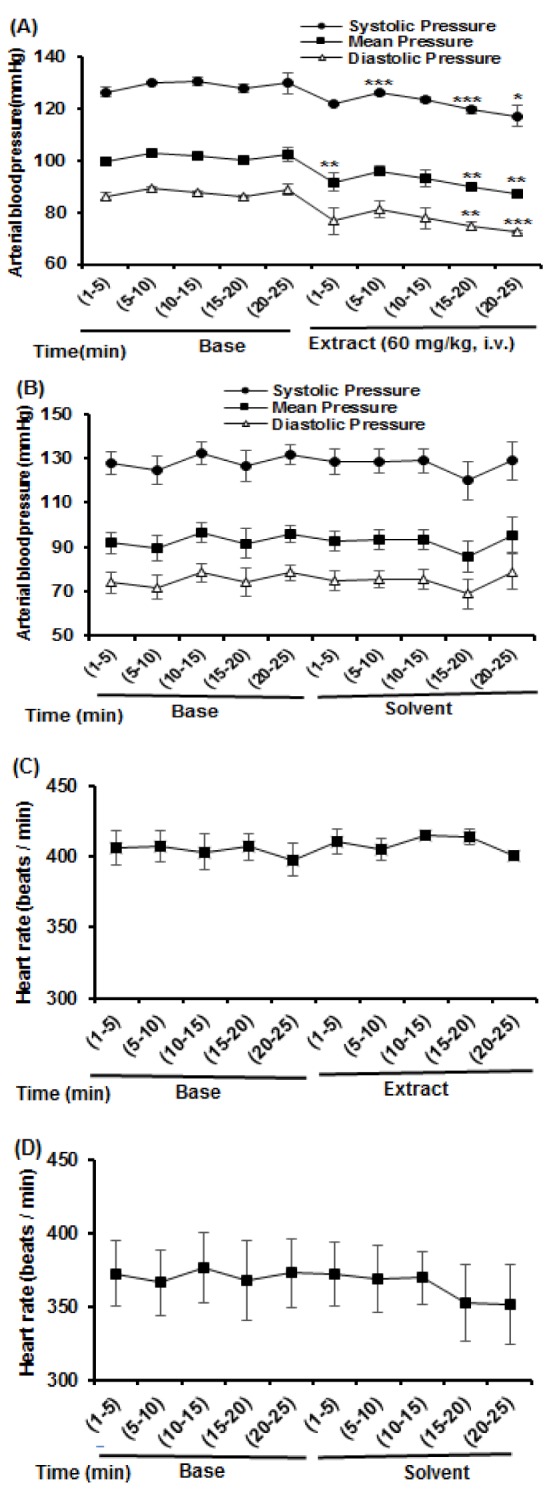
Effects of HEAE (60 mg/kg, i.v.) and its solvent (ethanol 70 %, i.v.) on arterial blood pressure and HR in anaesthetized rats. Effects of HEAE on blood pressure (A) and HR (C).Effects of solvent (equivalent volume of the HEAE) on blood pressure (B) and HR (D). Each value shows mean ± S.E.M. (n = 5 for each) and statistical analyses were performed by means of paired - sample t- test. ***p < 0.001, **p < 0.01, *p < 0.05 significantly different compared to same times in base stage.

The HEAE (60 mg/kg) decreased blood pressure (MAP, SBP, DBP), but baseline values of blood pressure, prior to and after i.v. administration of ethanol (as HEAE solvent) were not significantly different (Figure 2A, B(Fig. 2)). Neither HEAE nor ethanol altered HR during the observation period after treatment (Figure 2C, D(Fig. 2)). During the base stage before HEAE administration, the recorded MAP was constant ranging from 99 to 103 mm Hg in different periods of time (mins 1-25, n = 5). After administration of HEAE (60 mg/kg, i.v.), the MAP decreased to 91.8 ± 3.4 mm Hg in mins 1-5, to 96.1 ± 1.9 mm Hg in mins 5-10, to 93.1 ± 3.2 mm Hg in mins 10-15, to 89.8 ± 0.6 mm Hg in mins 15-20, and to 87.4 ± 1.7 in mins 20-25 (n = 5).

### Effects of HEAE and its solvent (ethanol 70 %) on arterial blood pressure and HR responses to ACh

**Figure 3:**
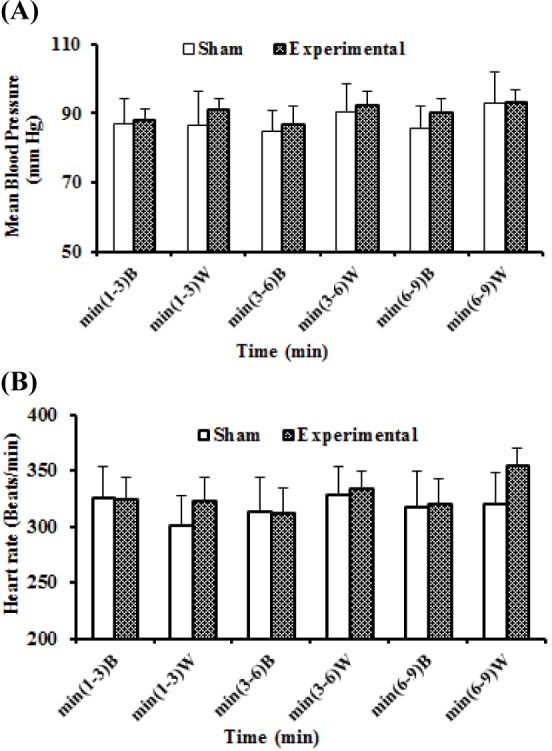
Alteration of MAP: curve A and HR: curve B in anaesthetized rats in base stage and after i.v. administration of distilled water in sham operated and experimental groups for cholinergic system. The “B” in X axis refers to the base stage that rats no treated, and “W” refers to the control drug stage, treated with distilled water as ACh solvent. The difference between same stages and same times in two groups were determined by means of unpaired t- test.

In sham operated and experimental animals, the mean values of basal and control drug stage (i.v. treatment with distilled water as ACh solvent) MAP and HR recorded were not significantly different (Figure 3, A: MAP, and B: HR(Fig. 3)).

**Figure 4:**
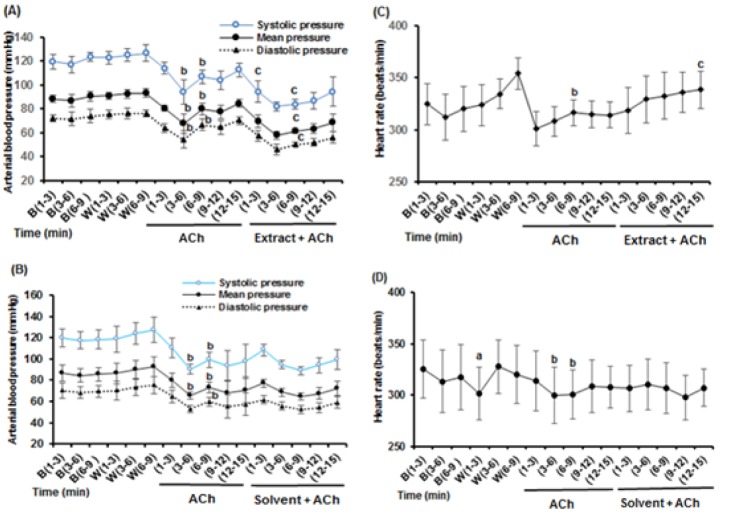
Hydroethanolic extract of *A. eriophora* (HEAE) and its solvent (ethanol 70 %) effects over arterial blood pressure and heart rate (HR) in anaesthetized rats in the presence of ACh (10 µg/kg). Change in blood pressure (A) and HR (C) with HEAE (60 mg/kg, i.v.) and ACh. Change in blood pressure (B) and HR (D) with solvent (equivalent volume of HEAE, i.v.) and ACh. The “B” refers to the base stage that rats no treated, and “W” refers to the control drug stage, treated with distilled water as ACh solvent. The difference between same times in different stages were determined by means of repeated measure.a p < 0.05 significantly different compared to B. bp < 0.05 significantly different compared to W; cp < 0.05 significantly different compared to ACh.

In both sham operated and experimental groups, i.v. injection of ACh decreased MAP, SBP, DBP and HR in anaesthetized rats (Figure 4(Fig. 4)). In sham operated group, i.v. injection of ACh decreased MAP, SBP and DBP in rats that were pretreated with ethanol, but effects were similar to the stage that ACh injected alone (Figure 4B(Fig. 4)). In experimental rats, ACh decreased MAP, SBP and DBP after administration of HEAE (60 mg/kg) and reduction of blood pressure was greater than the stage that ACh injected alone (Figure 4A(Fig. 4)). Heart rate in the presence of ethanol and ACh was similar with ACh alone (Figure 4D(Fig. 4)), however HR increased in the presence of HEAE and ACh (Figure 4C(Fig. 4)). Distilled water transitorily significant decreased HR in the first minutes in sham operated animals which progressively returned back to the basal value in about 2-3 min (Figure 4D(Fig. 4)).

### Effects of HEAE and its solvent (ethanol 70 %) on arterial blood pressure and HR responses to L-NAME

**Figure 5:**
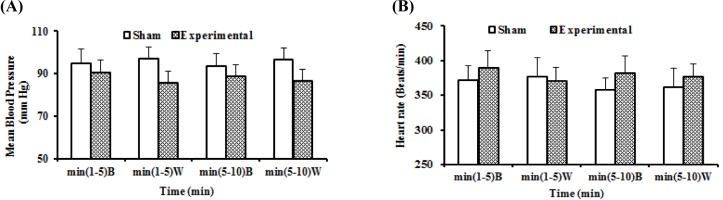
Change in MAP: curve A and HR: curve B in anaesthetized rats in baseline and after i.v. administration of distilled water in sham operated and experimental groups for nitrergic system. The “B” refers to the baseline that rats no treated, and “W” refers to the control drug stage, treated with distilled water as L-NAME solvent. The difference between same stages and same times in two groups were determined by means of unpaired t- test.

**Figure 6:**
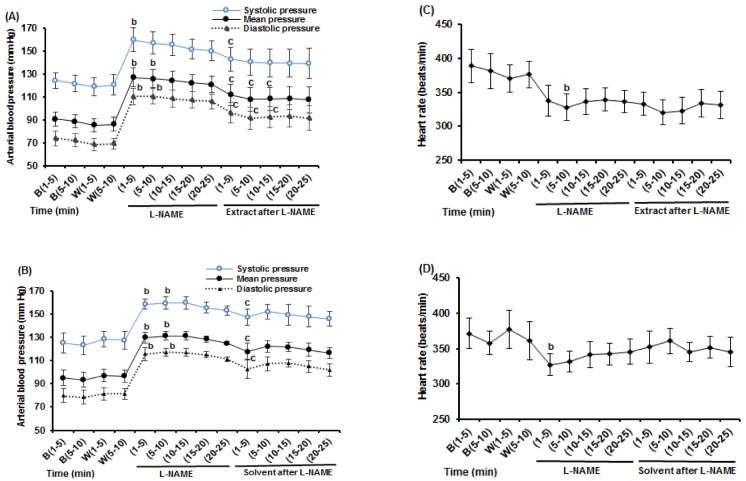
Hydroethanolic extract of *A. eriophora* (HEAE) and its solvent (ethanol 70 %) effects over arterial blood pressure and heart rate (HR) in anaesthetized rats after intravenous (i.v.) administration of L-NAME (5 mg/kg). Change in blood pressure (A) and HR (C) with HEAE (60 mg/kg, i.v.). Change in blood pressure (B) and HR (D) with solvent (equivalent volume of HEAE, i.v.). The “B” refers to the base stage that rats no treated, and “W” refers to the control drug stage, treated with distilled water as L-NAME solvent. The difference between same times in different stages were determined by means of repeated measure.b p < 0.05 significantly different compared to W; c p < 0.05 significantly different compared to L-NAME.

To identify the mechanisms involved in the HEAE-induced hypotension, the role of the endothelial signaling pathways were dissected. Because the NO-cGMP signalling is important in the regulation of endothelium-dependent vasorelaxation (Grange et al., 2001[11]; Queen and Ferro, 2006[22]), the effects of inhibition of endothelial NO - synthase (eNOS) activity were examined. In sham operated and experimental animals, the mean values of baseline and control drug stage (i.v. treatment with distilled water as L-NAME solvent) MAP and HR recorded were similar and have not significantly difference (Figure 5(Fig. 5), top: MAP, bottom HR). As shown in Figure 6(Fig. 6), i.v. administration of L-NAME (5 mg/kg), an inhibitor of eNOS, increased blood pressure (MAP, SBP, DBP) and decreased HR in sham operated and experimental groups. Data showed that i.v. administration of HEAE and ethanol decreased MAP in rats that pretreated with L-NAME (Figure 6A and B(Fig. 6)), but the decrease in MAP produced by HEAE (in experimental group) was significantly greater than solvent (in sham operated group). In experimental animals, the administration of L-NAME (5mg/kg, i.v.) to anaesthetized rats increased the MAP from 86.2 ± 5.8 mm Hg (n = 5) to a leveling point in 120.7 ± 7.1 mm Hg (n = 5). Administration of HEAE (60 mg/kg, i.v.) after the blood pressure levelling, caused a significant and long-lasting reduction in MAP values (Figure 6A(Fig. 6)).

Maximum hypotensive effects were observed within the 1-15 min after HEAE administration (compared with the same times in L-NAME treatment), while ethanol hypotensive effects in sham operated animals lasted shorter than in those observed in experimental rats. On the other hand, as shown in Figure 6C and 6D(Fig. 6), HR of both groups was not altered significantly by HEAE or ethanol. Injection of ethanol, after L-NAME in the first minutes, increased HR, but this effect was not statistically significant (Figure 6D(Fig. 6)). It is important to note that reduction in arterial blood pressure by HEAE after L-NAME is mainly due to reduction in diastolic pressure (Figure 6A(Fig. 6)).

## Discussion

The present study, using an *invivo* approach, shows for the first time that intravenous (i.v.) treatment of anaesthetized rats with extract obtained from leaves and flowers of *A. eriophora *lowers blood pressure. It has been reported that other *Achillea* species such as *A. millefolium* and *A. wilhelmsii *have beneficial effects on the cardiovascular system (Asgary et al., 2000[2]; Dall ҆ Acqua et al., 2011[5]; De Souza et al., 2011[7]). Nevertheless, the cardiovascular effects of *A. eriophora* have not ever been demonstrated. Interestingly, this study showed that i.v. administration of hydroethanolic extract of *A. eriophora *(HEAE) reduces the MAP of normal rats. According to our result, i.v. administration of 60 mg/kg of HEAE produced an intense and long-lasting hypotensive effect, without significant change in HR, but answer to this question that HEAE will be introduced as antihypertensive agent, further studies are required. Since hypertension is a chronic condition, so using medicinal plants for long-term use may be more beneficial (De Souza et al., 2011[7]; Xiong et al., 2014[29]).

There are a few studies dealing with the chemical composition of *A. eriophora* and its biological activity. The previous studies showed that 1,8-cineole, linalool, α-terpineole and geranylformate are the main components of the essential oil of *A. eriophora* (Ghasemi et al., 2008[10]). Some investigators (e.g. Dokhani et al., 2005[8]; De Souza et al., 2011[7]) have reported that polyphenolic compounds are present in different species of *Achillea* such as *A. eriophora*. 

Therefore, the hypotensive effects of HEAE can be attributed to a number of polyphenolic compounds such as flavonoids as hypotensive, vasorelaxant and antihypertensive agent (e.g. Ajay et al., 2003[1]; Jiang et al., 2005[13]; Morello et al., 2006[19]; Ajay et al., 2007[1]; Cho et al., 2007[3]; Magos et al., 2008[18]; Dong et al., 2009[9]; De Souza et al., 2011[7]).

In the present study, also an attempt was made to examine the role of cholinergic and nitrergic systems in the HEAE-induced hypotension. Result showed that treatment of animals with extract+ACh exhibited a significant decrease in MAP and tachycardia. The maximum hypotensive effect in the presence of extract +ACh was greater than ACh alone. These result suggest that blood pressure lowering effects of the extract is synergist with cholinergic system. It is important to note that the reduction of MAP in the presence of extract+ACh could initiated baroreceptor reflex that induced tachycardia (Lahlou et al., 2002[16]).

In this study, the i.v. administration of L-NAME as NO synthase inhibitor to normal rats increased the arterial blood pressure (SBP, DBP, MAP) and decreased HR that is consistent with previous studies (Hu et al., 1997[12]; Shin et al., 2014[24]). Injection of HEAE (60 mg/kg, i.v.) after L-NAME, caused a significant and persistent reduction in MAP and DBP with no alteration of HR. This hypotensive effect was more prolonged in L-NAME+HEAE treated animals than sham operated animals. These results indicate that the nitrergic system does not seem to play a significant role in the hypotensive effect of the extract. It should be remembered that in sham operated group, injection of ethanol as extract solvent after L-NAME in the first minutes induced a negligible and non-significant increase of HR. This effect is in agreement with previous study which demonstrated that ethanol, inhibits the bradycardic responses to NO synthase inhibitor (Wang and Pang, 1993[27]).

Since no changes in the HR were found in rats treated concomitantly with L-NAME and extract, it seems that the lowering of blood pressure may be due to reduction of total peripheral resistance. This hypothesis is supported by our result in which i.v. administration of extract significantly decreased DBP in L-NAME treated animals. It was proposed that lowering DBP induced by extract administration accompanied with tachycardia, but in contrast no change of HR was seen, so it can be said that extract may directly affect the heart.

The result of present study showed that HEAE has dual attenuate effects on blood pressure: via lowering total peripheral resistance and cardiac output, that may be synergist to cholinergic system and independent of nitrergic system. However, further studies will be required to elucidate the real mechanisms of this extract-induced hypotensive effect.

## Acknowledgement

The technical assistance of Esmaeil Khoshnam, and Zahra Hasanzadeh is acknowledged. This study was supported financial by the post-graduated grant No. SU9130527 from Shiraz University.
